# Cardiovascular diseases risk factors among recently arrived Eritrean refugees in Switzerland

**DOI:** 10.1186/s13104-019-4715-0

**Published:** 2019-10-21

**Authors:** Afona Chernet, Nicole Probst-Hensch, Véronique Sydow, Daniel H. Paris, Andreas Neumayr, Niklaus D. Labhardt

**Affiliations:** 1 Swiss Tropical and Public Health Institute, P.O. Box, 4002 Basel, Switzerland; 2 University of Basel, Basel, Switzerland; 3 Division of Infectious Diseases and Hospital Epidemiology, University Hospital Basel, Basel, Switzerland

**Keywords:** CVD risk factors, Eritrea, Migration, Refugees, Switzerland

## Abstract

**Objective:**

For the past 10 years, refugees from Eritrea represented the majority of asylum seekers in Switzerland. However, data on their health status remains limited. In this cross-sectional survey followed by a 1-year cohort study, we screened newly arrived Eritrean refugees for cardiovascular risk factors at arrival and 1-year post registration.

**Results:**

Among 107 participants (88.8% male; median age 25, 9 (9%) had a body mass index ≥ 25 kg/m^2^, one (1%) had elevated blood pressure, one (1%) had diabetes, 19% smoked and two (2%) had a low density lipoprotein (LDL) cholesterol ≥ 4.1 mmol/l. Among the 48 participants (5 females, 43 males) followed, there were no significant changes in cardiovascular risk profile 1 year post-arrival.

## Introduction

In the past decade high numbers of refugees arrived in Europe. Focus of research on migrants’ health in host countries has mainly been on communicable infectious diseases. Little attention was given to the prevalence of cardiovascular diseases (CVDs) and their risk factors. However, CVDs are known to be on the rise in many African countries, including major source countries of refugees arriving in Europe [1].

The few studies available report adaption of new life style among immigrants in host countries exposing them to a higher CVD risk [2]. A US study revealed at least one non-communicable disease in more than half of recently arrived adult refugees [3]. Frequently diagnosed CVD risk factors are; high body mass index (BMI), elevated blood pressure (BP), and smoking. In a study conducted in Canada, 30% of refugees screened had elevated BP [4]. Médecins sans Frontières reports a 20.9% prevalence of CVDs among Syrian refugees arriving in Jordan [5].

There is, however, nearly no data on CVD risk profile among African migrants arriving in Europe. Switzerland is one of the major destinations of Eritrean refugees. During the last decade, the majority of African migrants in Switzerland, accounting for almost 30%, were Eritrean origin [6]. In this cohort-study we report CVD risk profile among Eritrean refugees at arrival and its change 1 year post arrival in Switzerland.

## Main text

### Methods

In two Swiss cantons (Basel-Stadt, Basel-Land), we enrolled Eritrean refugees from February to November 2016. After a written consent to take part in the study, participants were screened for infectious and non-infectious diseases at baseline and thereafter followed for 12 months. Detailed recruitment procedure and results from infectious diseases screening has been described previously [7]. In this short communication we report from the same cohort the CVD risk profile at baseline and 1-year follow-up.

Study participants were eligible if they were aged 16 years and above, had arrived less than 1 year ago in Switzerland, had no physical complaints at recruitment and consented to the study. The recruitment period was driven by the study budget. We used a convenience sample-size including all individuals found eligible during the study-period.

For CVD risk profile screening, they were interviewed about life-style habits and smoking, thereafter, body-weight and height, waist circumference and blood pressure were measured. Thereafter venous blood was collected at fasting state for measurement of glycated hemoglobin (HbA1c) and lipid-panel [total cholesterol (TC), high density lipoprotein (HDL), low density lipoprotein (LDL), and triglycerides (TG)]. Screening was done at baseline and participants were re-contacted 1 year later for follow-up.

Data were collected on paper format and subsequently entered into EpiInfo version 7 (CDC, 1600 Clifton Road, Atlanta, USA). Statistical analyses were performed in Stata version 13 (StataCorp LP, 4905 Lakeway Drive, College Station, USA).

The study protocol was approved by the institutional research commission of the Swiss Tropical and Public Health Institute (Swiss TPH, Basel, Switzerland; reference no. FK 120; approval date: June 24, 2015) and the Ethics Committee of Northwest and Central Switzerland (reference no. EKNZ 2015-353; approval date: November 20, 2015). Participation was voluntary and people could withdraw from the study at any time without further obligations.

### Results

A total of 107 (11.2% female) refugees were enrolled at baseline. Median age was 25 (interquartile range [IQR] 20–28) years. Table 1 presents prevalence of CVDs risk factors at baseline. Generally, participants had a favourable CVD risk profile at arrival. Overall, 48 (45%) attended the 1-year follow-up visit. The remaining had either moved to a different canton or outside Switzerland (45%) or declined participation at follow-up visit (10%). Table 2 compares baseline and follow-up measures among the 48 participants with complete data. There was no significant change in median blood pressure and cholesterol values at 1 year follow-up. However, BMI and HbA1c showed a very small, but significant trend of change.

**Table 1:** Cardio-vascular risk factors among newly arrived Eritrean refugees in Switzerland

Measures	Variable (unit)	Categories	N (%)	Median (IQR)	
Age	Age (years)		107 (100.0)	25	20–28
Smoking	Smoking	Non-smoker	83 (77.6)	NA	NA
		Ex-smoker	4 (3.7)	NA	NA
		Smoker	20 (18.7)	NA	NA
Body mass index	BMI (kg/m^2^)	Over all	107 (100.0)	21.2	19.4–23.1
		< 18.5	15 (14.0)	17.1	16.8–18.2
		≥ 18.5 and ≤ 24.9	83 (77.6)	21.7	20.1–22.9
		≥ 25.0	9 (8.6)	27.2	26.5–28.1
Blood pressure	SBP (mmHg)	Over all		117	109–122
		< 120	67 (62.6)	112	103–116
		≥ 120 and ≤ 139	39 (36.4)	123	121–129
		≥ 140	1 (0.9)	140	NA
	DBP (mmHg)	Over all		70	64–77
		< 80	93 (86.9)	68	63–73
		≥ 80 and ≤ 89	13 (12.1)	83	81–87
		≥ 90	1 (0.9)	99	NA
Glycated hemoglobin	HbA1c (%)	Over all		5.0	4.8–5.2
		< 5.6	99 (92.5)	4.9	4.7–5.1
		≥ 5.6 and < 6.5	7 (6.5)	5.7	5.7–5.9
		≥ 6.5	1 (0.9)	6.6	NA
Lipid panel	TC (mmol/L)	Over all		3.9	3.3–4.5
		< 5.16	99 (92.5)	3.8	3.1–4.2
		≥ 5.16 and < 6.20	5 (4.7)	5.6	5.4–5.7
		≥ 6.20	3 (2.8)	6.8	6.5–6.8
	LDL (mmol/L)	Over all		2.2	1.8-2.7
		< 3.35	100 (93.5)	2.1	1.5–2.5
		≥ 3.35 and < 4.10	5 (4.7)	3.5	3.5–3.7
		≥ 4.10	2 (1.9)	4.6	4.4–4.9
	HDL (mmol/L)	Over all		1.2	1.0–1.4
		< 3.0	107 (100.0)	1.1	1.0–1.4
		≥ 3.0	0 (0.0)	NA	NA
	Triglycerides (mmol/L)	Over all		0.9	0.7–1.3
		< 2.26	104 (97.2)	1.0	0.7–1.2
		≥ 2.26 and < 4.50	3 (2.8)	2.9	2.4–3.1
		≥ 4.50	0 (0.0)	NA	NA
	TC to HDL ratio	< 3.0	45 (42.1)	2.5	2.1–2.7
		≥ 3.0	62 (57.9)	3.6	3.3–4.3

*IQR* interquartile range, *NA* not applicable

**Table 2:** Trend of lipid panel change at base-line and 1 year follow-up among recently migrated Eritrean refugees to Switzerland

Parameters	Categories	Base-line			Follow-up			N = 48
		N (%)	Median (IQR)		N (%)	Median (IQR)		Statistics
BMI								
Body mass index (kg/m^2^)	Over all	48 (100.0)	21.7	19.2–23.1	48 (100.0)	22.2	19.2–23.1	t(47) = − 2.56; p = 0.01
	< 18.5	7 (14.6)	17.5	16.3–18.3	5 (10.4)	17.5	16.8–18.2	
	≥ 18.5 and ≤ 24.9	34 (70.8)	21.7	20.1–22.6	36 (75.0)	22.0	21.0–23.1	
	≥ 25.0	7 (14.6)	27.2	26.5–28.3	7 (14.6)	27.9	25.6–28.3	
Blood pressure								
Systolic blood pressure (mmHg)	Over all	48 (100.0)	115.2	108.5–120.5	48 (100.0)	112.5	108.5–120.0	t(47) = 1.15; p = 0.26
	< 120	32 (66.7)	112.0	104.0–115.5	35 (72.9)	111.0	106.0–114.0	
	≥ 120 and ≤ 139	16 (33.3)	124.5	120.5–130.5	13 (27.1)	124.0	123.0–126.0	
	≥ 140	0			0			
Diastolic blood pressure (mmHg)	Over all	48 (100.0)	71.0	64.0–77.0	48 (100.0)	68	63.0–72.5	t(47) = 1.43; p = 0.16
	< 80	46 (95.8)	70.5	64.0–76.0	45 (93.8)	66	63.0–71.0	
	≥ 80 and ≤ 89	2 (4.2)	83.5	80.0–87.0	3 (6.2)	81	81.0–84.0	
	≥ 90	0			0			
HbA1c								
Glycated hemoglobin (%)	Over all	48 (100.0)	5.0	4.8–5.1	48 (100.0)	5.0	5.0–5.2	t(47) = − 3.44; p = 0.001
	< 5.6	45 (93.8)	5.0	4.8–5.1	45 (93.8)	5.0	5.0–5.2	
	≥ 5.6 and < 6.5	3 (6.2)	5.7	5.6–5.8	3 (6.2)	5.8	5.7–6.1	
	≥ 6.5	0			0			
Cholesterol								
Total Cholesterol [TC] (mmol/L)	Over all	48 (100.0)	4.0	3.3–4.6	48 (100.0)	4.0	3.4–4.6	t(47) = 0.80; p = 0.43
	<5.16	42 (87.5)	3.9	3.2–4.4	43 (89.6)	3.9	3.3–4.3	
	≥ 5.16 and < 6.20	4 (8.3)	5.5	5.4–5.8	5 (10.4)	5.4	5.3–5.4	
	≥ 6.20	2 (4.2)	6.7	6.5–6.8	0			
Low density lipoprotein [LDL] (mmol/L)	Over all	48 (100.0)	2.3	1.8–3.0	48 (100.0)	2.2	1.7–2.5	t(47) = 1.94; p = 0.06
	< 3.35	43 (89.6)	2.1	1.7–2.5	45 (93.8)	2.1	1.6–2.4	
	≥ 3.35 and < 4.10	4 (8.3)	3.5	3.5–3.7	3 (6.2)	3.9	3.5–3.9	
	≥ 4.10	1 (2.1)	4.4	NA	0			
High density lipoprotein [HDL] (mmol/L)	Over all	48 (100.0)	1.2	1.0–1.5	48 (100.0)	1.3	1.1–1.4	t(47) = 0.11; p = 0.92
	< 3.0	48 (100.0)	1.2	1.0–1.5	48 (100.0)	1.3	1.1–1.4	
	≥ 3.0	0			0			
Triglycerides (mmol/L)	Over all	48 (100.0)	1.0	0.8–1.4	48 (100.0)	1.0	0.8–1.4	t(47) = − 1.59; p = 0.12
	< 2.26	48 (100.0)	1.0	0.8–1.4	43 (89.6)	1.0	0.8–1.2	
	≥ 2.26 and < 4.50	0			4 (8.3)	2.6	2.3–3.0	
	≥ 4.50	0			1 (2.1)	5.3	NA	
TC to HDL ratio	Over all	48 (100.0)	3.2	2.8–3.8	48 (100.0)	3.0	2.8–3.7	t(47) = 1.08; p = 0.29
	< 3.0	18 (37.5)	2.6	2.4–2.9	21 (43.8)	2.8	2.5–2.9	
	≥ 3.0	30 (62.5)	3.7	3.2–4.4	27 (56.2)	3.5	3.2–4.2	

*IQR* interquartile range, *NA* not applicable

### Discussion

In this cohort study Eritrean refugees had a very favourable CVD risk profile at arrival in Switzerland and CVD risk factors did not show any relevant change 1 year post arrival in the host country. This study has several limitations. First, most study participants were in their young age (median 24 years) where generally the CVD risk profile is low. Second, only half of participants could be followed at 1 year due to high numbers of participants moving to different areas in Switzerland or Europe. Third, the follow-up period of 1-year was rather short.

In summary, our data are in line with survey data from Eritrea where the CVD risk profile in the general population was very low [8]. In our study, young Eritrean refugees had a very favorable CVD risk profile and showed no relevant increase in body-weight, cholesterol levels, blood pressure or blood glucose 1 year after arrival in Switzerland.

## Limitations

- Small sample size of participants.
- Relatively low participation of female refugees.
- Short follow-up time.

## Data Availability

Not applicable.

## Abbreviations

BMI: body mass index
CVDs: cardiovascular diseases
HbA1c: glycated hemoglobin
HDL: high density lipoprotein
LDL: low density lipoprotein
TC: total cholesterol
TG: triglycerides

## References

[1] WHO: Global Status Report on NCDs. WHO. 2010. http://www.who.int/chp/ncd_global_status_report/en/. Accessed 21 Feb 2018.

[2] Jen KLC, Jamil H, Zhou K, Breejen K, Arnetz BB (2017). Sex differences and predictors of changes in body weight and noncommunicable diseases in a random, newly-arrived group of refugees followed for two years. J Immigr Minor Health..

[3] Yun K, Hebrank K, Graber LK, Sullivan M-C, Chen I, Gupta J (2012). High prevalence of chronic non-communicable conditions among adult refugees: implications for practice and policy. J Community Health.

[4] Redditt VJ, Graziano D, Janakiram P, Rashid M (2015). Health status of newly arrived refugees in Toronto, Ont: part 2: chronic diseases. Can Fam Physician Med Fam Can..

[5] Collins DRJ, Jobanputra K, Frost T, Muhammed S, Ward A, Shafei AA (2017). Cardiovascular disease risk and prevention amongst Syrian refugees: mixed methods study of Médecins Sans Frontières programme in Jordan. Confl Health..

[6] Staatssekretariat für Migration, SEM. Asylstatistik. 2017. https://www.sem.admin.ch/sem/de/home/publiservice/statistik/asylstatistik/archiv/2017/12.html. Accessed 2 Feb 2018.

[7] Chernet A, Neumayr A, Hatz C, Kling K, Sydow V, Rentsch K (2017). Spectrum of infectious diseases among newly arrived Eritrean refugees in Switzerland: a cross-sectional study. Int J Public Health..

[8] WHO: Global Database on Body Mass Index. 2004. http://apps.who.int/bmi/index.jsp?introPage=intro_3.html. Accessed 30 Jan 2018.

